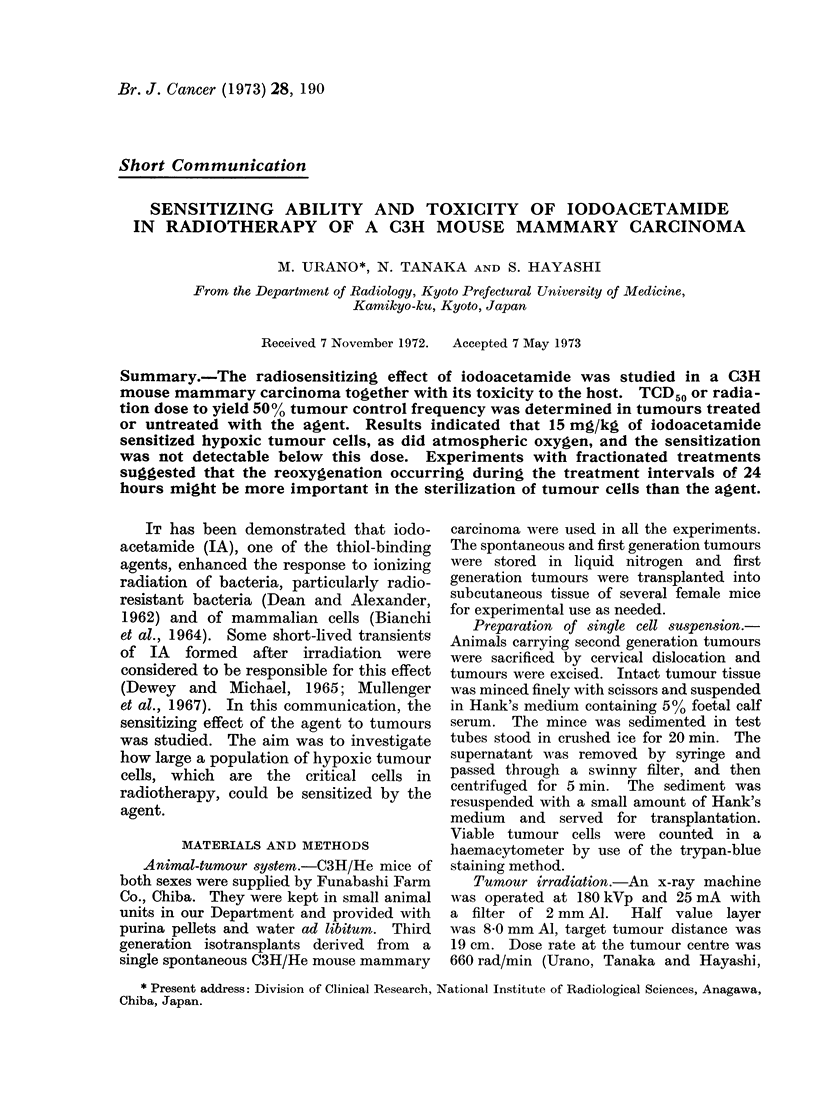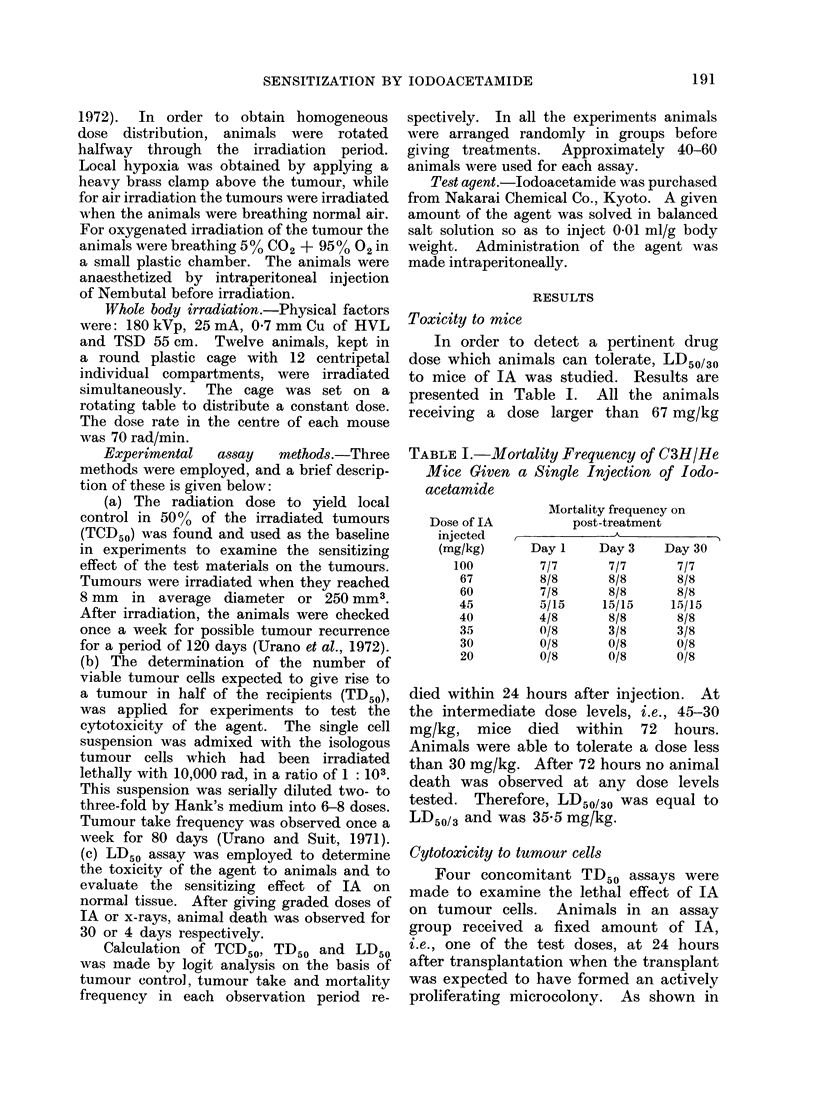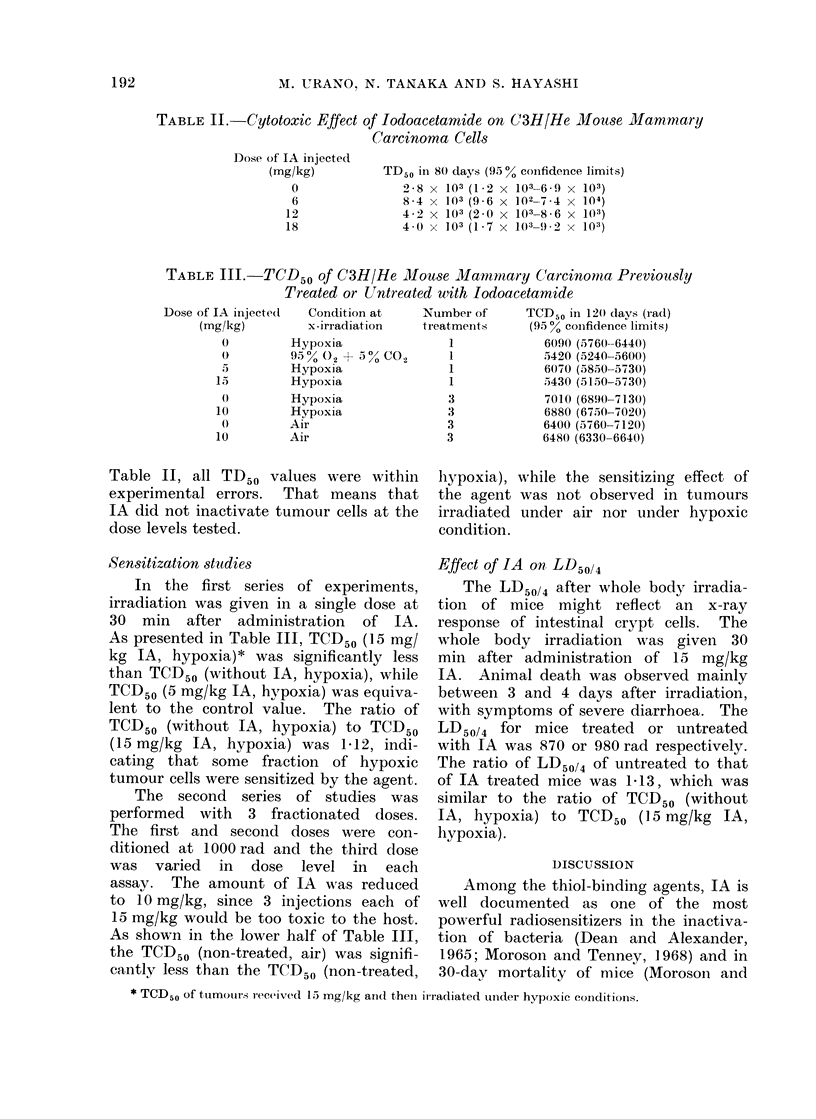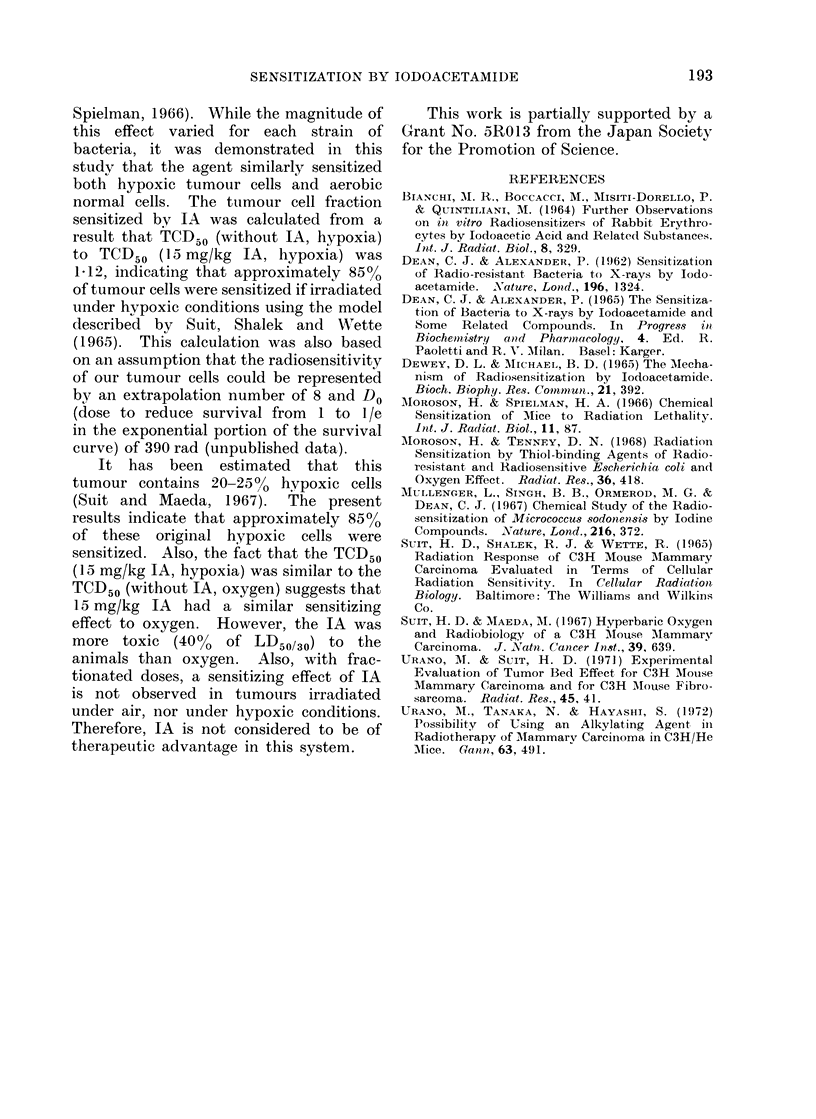# Sensitizing Ability and Toxicity of Iodoacetamide in Radiotherapy of a C3H Mouse Mammary Carcinoma

**DOI:** 10.1038/bjc.1973.136

**Published:** 1973-08

**Authors:** M. Urano, N. Tanaka, S. Hayashi

## Abstract

The radiosensitizing effect of iodoacetamide was studied in a C3H mouse mammary carcinoma together with its toxicity to the host. TCD_50_ or radiation dose to yield 50% tumour control frequency was determined in tumours treated or untreated with the agent. Results indicated that 15 mg/kg of iodoacetamide sensitized hypoxic tumour cells, as did atmospheric oxygen, and the sensitization was not detectable below this dose. Experiments with fractionated treatments suggested that the reoxygenation occurring during the treatment intervals of 24 hours might be more important in the sterilization of tumour cells than the agent.


*Br. J. Cancer* (1973) **28**, 190

### *Short Communication*

### SENSITIZING ABILITY AND TOXICITY OF IODOACETAMIDE IN RADIOTHERAPY OF A C3H MOUSE MAMMARY CARCINOMA

M. URANO*, N. TANAKA AND S. HAYASHI

*From the Department of Radiology, Kyoto Prefectural University of Medicine, Kamikyo-ku, Kyoto, Japan*

Received 7 November 1972. Accepted 7 May 1973

**Summary.—The radiosensitizing effect of iodoacetamide was studied in a C3H mouse mammary carcinoma together with its toxicity to the host. $TCD_{50}$ or radiation dose to yield 50% tumour control frequency was determined in tumours treated or untreated with the agent. Results indicated that 15 mg/kg of iodoacetamide sensitized hypoxic tumour cells, as did atmospheric oxygen, and the sensitization was not detectable below this dose. Experiments with fractionated treatments suggested that the reoxygenation occurring during the treatment intervals of 24 hours might be more important in the sterilization of tumour cells than the agent.**

IT has been demonstrated that iodoacetamide (IA), one of the thiol-binding agents, enhanced the response to ionizing radiation of bacteria, particularly radioresistant bacteria (Dean and Alexander, 1962) and of mammalian cells (Bianchi *et al.*, 1964). Some short-lived transients of IA formed after irradiation were considered to be responsible for this effect (Dewey and Michael, 1965; Mullenger *et al.*, 1967). In this communication, the sensitizing effect of the agent to tumours was studied. The aim was to investigate how large a population of hypoxic tumour cells, which are the critical cells in radiotherapy, could be sensitized by the agent.

### MATERIALS AND METHODS

*Animal-tumour system.*—C3H/He mice of both sexes were supplied by Funabashi Farm Co., Chiba. They were kept in small animal units in our Department and provided with purina pellets and water *ad libitum*. Third generation isotransplants derived from a single spontaneous C3H/He mouse mammary carcinoma were used in all the experiments. The spontaneous and first generation tumours were stored in liquid nitrogen and first generation tumours were transplanted into subcutaneous tissue of several female mice for experimental use as needed.

*Preparation of single cell suspension.*—Animals carrying second generation tumours were sacrificed by cervical dislocation and tumours were excised. Intact tumour tissue was minced finely with scissors and suspended in Hank's medium containing 5% foetal calf serum. The mince was sedimented in test tubes stood in crushed ice for 20 min. The supernatant was removed by syringe and passed through a swinny filter, and then centrifuged for 5 min. The sediment was resuspended with a small amount of Hank's medium and served for transplantation. Viable tumour cells were counted in a haemacytometer by use of the trypan-blue staining method.

*Tumour irradiation.*—An x-ray machine was operated at 180 kVp and 25 mA with a filter of 2 mm Al. Half value layer was 8·0 mm Al, target tumour distance was 19 cm. Dose rate at the tumour centre was 660 rad/min (Urano, Tanaka and Hayashi,

* Present address: Division of Clinical Research, National Institute of Radiological Sciences, Anagawa, Chiba, Japan.

1972). In order to obtain homogeneous dose distribution, animals were rotated halfway through the irradiation period. Local hypoxia was obtained by applying a heavy brass clamp above the tumour, while for air irradiation the tumours were irradiated when the animals were breathing normal air. For oxygenated irradiation of the tumour the animals were breathing 5% $CO_2$ + 95% $O_2$ in a small plastic chamber. The animals were anaesthetized by intraperitoneal injection of Nembutal before irradiation.

*Whole body irradiation.*—Physical factors were: 180 kVp, 25 mA, 0·7 mm Cu of HVL and TSD 55 cm. Twelve animals, kept in a round plastic cage with 12 centripetal individual compartments, were irradiated simultaneously. The cage was set on a rotating table to distribute a constant dose. The dose rate in the centre of each mouse was 70 rad/min.

*Experimental assay methods.*—Three methods were employed, and a brief description of these is given below:

(a) The radiation dose to yield local control in 50% of the irradiated tumours ($TCD_{50}$) was found and used as the baseline in experiments to examine the sensitizing effect of the test materials on the tumours. Tumours were irradiated when they reached 8 mm in average diameter or 250 mm$^3$. After irradiation, the animals were checked once a week for possible tumour recurrence for a period of 120 days (Urano *et al.*, 1972).
(b) The determination of the number of viable tumour cells expected to give rise to a tumour in half of the recipients ($TD_{50}$), was applied for experiments to test the cytotoxicity of the agent. The single cell suspension was admixed with the isologous tumour cells which had been irradiated lethally with 10,000 rad, in a ratio of 1 : $10^3$. This suspension was serially diluted two- to three-fold by Hank's medium into 6–8 doses. Tumour take frequency was observed once a week for 80 days (Urano and Suit, 1971).
(c) $LD_{50}$ assay was employed to determine the toxicity of the agent to animals and to evaluate the sensitizing effect of IA on normal tissue. After giving graded doses of IA or x-rays, animal death was observed for 30 or 4 days respectively.

Calculation of $TCD_{50}$, $TD_{50}$ and $LD_{50}$ was made by logit analysis on the basis of tumour control, tumour take and mortality frequency in each observation period respectively. In all the experiments animals were arranged randomly in groups before giving treatments. Approximately 40–60 animals were used for each assay.

*Test agent.*—Iodoacetamide was purchased from Nakarai Chemical Co., Kyoto. A given amount of the agent was solved in balanced salt solution so as to inject 0·01 ml/g body weight. Administration of the agent was made intraperitoneally.

### RESULTS

### *Toxicity to mice*

In order to detect a pertinent drug dose which animals can tolerate, $LD_{50/30}$ to mice of IA was studied. Results are presented in Table I. All the animals receiving a dose larger than 67 mg/kg died within 24 hours after injection. At the intermediate dose levels, *i.e.*, 45–30 mg/kg, mice died within 72 hours. Animals were able to tolerate a dose less than 30 mg/kg. After 72 hours no animal death was observed at any dose levels tested. Therefore, $LD_{50/30}$ was equal to $LD_{50/3}$ and was 35·5 mg/kg.

TABLE I.—*Mortality Frequency of C3H/He Mice Given a Single Injection of Iodoacetamide*

| Dose of IA injected (mg/kg) | Mortality frequency on post-treatment Day 1 | Day 3 | Day 30 |
|---|---|---|---|
| 100 | 7/7 | 7/7 | 7/7 |
| 67 | 8/8 | 8/8 | 8/8 |
| 60 | 7/8 | 8/8 | 8/8 |
| 45 | 5/15 | 15/15 | 15/15 |
| 40 | 4/8 | 8/8 | 8/8 |
| 35 | 0/8 | 3/8 | 3/8 |
| 30 | 0/8 | 0/8 | 0/8 |
| 20 | 0/8 | 0/8 | 0/8 |

### *Cytotoxicity to tumour cells*

Four concomitant $TD_{50}$ assays were made to examine the lethal effect of IA on tumour cells. Animals in an assay group received a fixed amount of IA, *i.e.*, one of the test doses, at 24 hours after transplantation when the transplant was expected to have formed an actively proliferating microcolony. As shown in

TABLE II.—*Cytotoxic Effect of Iodoacetamide on C3H/He Mouse Mammary Carcinoma Cells*

| Dose of IA injected (mg/kg) | $TD_{50}$ in 80 days (95% confidence limits) |
|---|---|
| 0 | $2 \cdot 8 \times 10^3$ ($1 \cdot 2 \times 10^3$–$6 \cdot 9 \times 10^3$) |
| 6 | $8 \cdot 4 \times 10^3$ ($9 \cdot 6 \times 10^2$–$7 \cdot 4 \times 10^4$) |
| 12 | $4 \cdot 2 \times 10^3$ ($2 \cdot 0 \times 10^3$–$8 \cdot 6 \times 10^3$) |
| 18 | $4 \cdot 0 \times 10^3$ ($1 \cdot 7 \times 10^3$–$9 \cdot 2 \times 10^3$) |

TABLE III.—*$TCD_{50}$ of C3H/He Mouse Mammary Carcinoma Previously Treated or Untreated with Iodoacetamide*

| Dose of IA injected (mg/kg) | Condition at x-irradiation | Number of treatments | $TCD_{50}$ in 120 days (rad) (95% confidence limits) |
|---|---|---|---|
| 0 | Hypoxia | 1 | 6090 (5760–6440) |
| 0 | 95% $O_2$ + 5% $CO_2$ | 1 | 5420 (5240–5600) |
| 5 | Hypoxia | 1 | 6070 (5850–5730) |
| 15 | Hypoxia | 1 | 5430 (5150–5730) |
| 0 | Hypoxia | 3 | 7010 (6890–7130) |
| 10 | Hypoxia | 3 | 6880 (6750–7020) |
| 0 | Air | 3 | 6400 (5760–7120) |
| 10 | Air | 3 | 6480 (6330–6640) |

Table II, all $TD_{50}$ values were within experimental errors. That means that IA did not inactivate tumour cells at the dose levels tested.

*Sensitization studies*

In the first series of experiments, irradiation was given in a single dose at 30 min after administration of IA. As presented in Table III, $TCD_{50}$ (15 mg/kg IA, hypoxia)* was significantly less than $TCD_{50}$ (without IA, hypoxia), while $TCD_{50}$ (5 mg/kg IA, hypoxia) was equivalent to the control value. The ratio of $TCD_{50}$ (without IA, hypoxia) to $TCD_{50}$ (15 mg/kg IA, hypoxia) was 1·12, indicating that some fraction of hypoxic tumour cells were sensitized by the agent.

The second series of studies was performed with 3 fractionated doses. The first and second doses were conditioned at 1000 rad and the third dose was varied in dose level in each assay. The amount of IA was reduced to 10 mg/kg, since 3 injections each of 15 mg/kg would be too toxic to the host. As shown in the lower half of Table III, the $TCD_{50}$ (non-treated, air) was significantly less than the $TCD_{50}$ (non-treated, hypoxia), while the sensitizing effect of the agent was not observed in tumours irradiated under air nor under hypoxic condition.

*Effect of IA on $LD_{50/4}$*

The $LD_{50/4}$ after whole body irradiation of mice might reflect an x-ray response of intestinal crypt cells. The whole body irradiation was given 30 min after administration of 15 mg/kg IA. Animal death was observed mainly between 3 and 4 days after irradiation, with symptoms of severe diarrhoea. The $LD_{50/4}$ for mice treated or untreated with IA was 870 or 980 rad respectively. The ratio of $LD_{50/4}$ of untreated to that of IA treated mice was 1·13, which was similar to the ratio of $TCD_{50}$ (without IA, hypoxia) to $TCD_{50}$ (15 mg/kg IA, hypoxia).

### DISCUSSION

Among the thiol-binding agents, IA is well documented as one of the most powerful radiosensitizers in the inactivation of bacteria (Dean and Alexander, 1965; Moroson and Tenney, 1968) and in 30-day mortality of mice (Moroson and

* $TCD_{50}$ of tumours received 15 mg/kg and then irradiated under hypoxic conditions.

Spielman, 1966). While the magnitude of this effect varied for each strain of bacteria, it was demonstrated in this study that the agent similarly sensitized both hypoxic tumour cells and aerobic normal cells. The tumour cell fraction sensitized by IA was calculated from a result that $TCD_{50}$ (without IA, hypoxia) to $TCD_{50}$ (15 mg/kg IA, hypoxia) was 1·12, indicating that approximately 85% of tumour cells were sensitized if irradiated under hypoxic conditions using the model described by Suit, Shalek and Wette (1965). This calculation was also based on an assumption that the radiosensitivity of our tumour cells could be represented by an extrapolation number of 8 and $D_0$ (dose to reduce survival from 1 to 1/e in the exponential portion of the survival curve) of 390 rad (unpublished data).

It has been estimated that this tumour contains 20–25% hypoxic cells (Suit and Maeda, 1967). The present results indicate that approximately 85% of these original hypoxic cells were sensitized. Also, the fact that the $TCD_{50}$ (15 mg/kg IA, hypoxia) was similar to the $TCD_{50}$ (without IA, oxygen) suggests that 15 mg/kg IA had a similar sensitizing effect to oxygen. However, the IA was more toxic (40% of $LD_{50/30}$) to the animals than oxygen. Also, with fractionated doses, a sensitizing effect of IA is not observed in tumours irradiated under air, nor under hypoxic conditions. Therefore, IA is not considered to be of therapeutic advantage in this system.

This work is partially supported by a Grant No. 5R013 from the Japan Society for the Promotion of Science.

### REFERENCES

BIANCHI, M. R., BOCCACCI, M., MISITI-DORELLO, P. & QUINTILIANI, M. (1964) Further Observations on *in vitro* Radiosensitizers of Rabbit Erythrocytes by Iodoacetic Acid and Related Substances. *Int. J. Radiat. Biol.*, **8**, 329.

DEAN, C. J. & ALEXANDER, P. (1962) Sensitization of Radio-resistant Bacteria to X-rays by Iodoacetamide. *Nature, Lond.*, **196**, 1324.

DEAN, C. J. & ALEXANDER, P. (1965) The Sensitization of Bacteria to X-rays by Iodoacetamide and Some Related Compounds. In *Progress in Biochemistry and Pharmacology*, **4**. Ed. R. Paoletti and R. V. Milan. Basel: Karger.

DEWEY, D. L. & MICHAEL, B. D. (1965) The Mechanism of Radiosensitization by Iodoacetamide. *Bioch. Biophy. Res. Commun.*, **21**, 392.

MOROSON, H. & SPIELMAN, H. A. (1966) Chemical Sensitization of Mice to Radiation Lethality. *Int. J. Radiat. Biol.*, **11**, 87.

MOROSON, H. & TENNEY, D. N. (1968) Radiation Sensitization by Thiol-binding Agents of Radioresistant and Radiosensitive *Escherichia coli* and Oxygen Effect. *Radiat. Res.*, **36**, 418.

MULLENGER, L., SINGH, B. B., ORMEROD, M. G. & DEAN, C. J. (1967) Chemical Study of the Radiosensitization of *Micrococcus sodonensis* by Iodine Compounds. *Nature, Lond.*, **216**, 372.

SUIT, H. D., SHALEK, R. J. & WETTE, R. (1965) Radiation Response of C3H Mouse Mammary Carcinoma Evaluated in Terms of Cellular Radiation Sensitivity. In *Cellular Radiation Biology*. Baltimore: The Williams and Wilkins Co.

SUIT, H. D. & MAEDA, M. (1967) Hyperbaric Oxygen and Radiobiology of a C3H Mouse Mammary Carcinoma. *J. Natn. Cancer Inst.*, **39**, 639.

URANO, M. & SUIT, H. D. (1971) Experimental Evaluation of Tumor Bed Effect for C3H Mouse Mammary Carcinoma and for C3H Mouse Fibrosarcoma. *Radiat. Res.*, **45**, 41.

URANO, M., TANAKA, N. & HAYASHI, S. (1972) Possibility of Using an Alkylating Agent in Radiotherapy of Mammary Carcinoma in C3H/He Mice. *Gann*, **63**, 491.